# Comparing personal insight gains due to consideration of a recent dream and consideration of a recent event using the Ullman and Schredl dream group methods

**DOI:** 10.3389/fpsyg.2015.00831

**Published:** 2015-06-18

**Authors:** Christopher L. Edwards, Josie E. Malinowski, Shauna L. McGee, Paul D. Bennett, Perrine M. Ruby, Mark T. Blagrove

**Affiliations:** ^1^Sleep Laboratory, Department of Psychology, Swansea University, Swansea, UK; ^2^Department of Psychology, Swansea University, Swansea, UK; ^3^Department of Psychology, University of Bedfordshire, Luton, UK; ^4^Brain Dynamics and Cognition Team, Lyon Neuroscience Research Centre, INSERM U1028, Lyon, France

**Keywords:** dreams, insight, sleep, REM sleep, psychotherapy, psychopathology

## Abstract

There have been reports and claims in the psychotherapeutic literature that the consideration of recent dreams can result in personal realizations and insight. There is theoretical support for these claims from work on rapid eye movement (REM) sleep having a function of the consolidation of emotional memories and the creative formation of connections between new and older memories. To investigate these claims, 11 participants (10 females, one male) reported and considered a recent home dream in a dream discussion group that following the “Appreciating dreams” method of Montague Ullman. The group ran 11 times, each participant attending and participating once. A further nine participants (seven females, two males) reported and considered a recent home dream in a group that followed the “Listening to the dreamer” method of Michael Schredl. The two studies each had a control condition where the participant also reported a recent event, the consideration of which followed the same technique as was followed for the dream report. Outcomes of the discussions were assessed by the participants on the Gains from Dream Interpretation (GDI) scale, and on its counterpart, the Gains from Event Interpretation scale. High ratings on the GDI experiential-insight subscale were reported for both methods, when applied to dreams, and for the Ullman method Exploration-Insight ratings for the dream condition were significantly higher than for the control event condition. In the Ullman method, self-assessment of personal insight due to consideration of dream content was also significantly higher than for the event consideration condition. The findings support the view that benefits can be obtained from the consideration of dream content, in terms of identifying the waking life sources of dream content, and because personal insight may also occur. To investigate the mechanisms for the findings, the studies should be repeated with REM and non-REM dream reports, hypothesizing greater insight from the former.

## Introduction

There have been reports and claims in the psychotherapeutic and psychoanalytic literature that the consideration of recent dreams can result in personal realizations and insight (e.g., Freud, 1953; Blechner, 2001; Blass, 2002; Lippmann, 2002). Edwards et al. (2013) addressed these claims in a non-clinical dream interpretation group that followed the dream group method of Montague Ullman (1996). Self-ratings of gains from participation in the dream group sessions, assessed by the Gains from Dream Interpretation (GDI) questionnaire, showed that the Ullman technique is an effective procedure for establishing connections between dream content and recent waking life experiences, even though wake life sources were found for only 14% of dream report text. The mean Exploration-Insight subscale score on the GDI questionnaire was very high (8.17 on a scale from 1 to 9) and comparable to outcomes on the same measure from sessions using the well-established Hill (1996) therapist-led dream interpretation method. The current study follows the method used in the Ullman dream group study of Edwards et al. (2013) but includes also as a control condition the consideration of a report of a recent personally significant event, using the same interpretation technique as for the recent dream. A second technique for considering dreams, that of Schredl (2007, 2011), is also used for a separate sample of participants, again with an event interpretation control condition.

There are theoretical and empirical reasons to support the proposal that salient waking life events and concerns may appear in dreams and that the consideration of that dream content may result in realizations or insight about one’s life circumstances. Edwards et al. (2013) review evidence that dreams reflect the current waking life concerns or recent personally significant waking life events of the dreamer. This reflection follows from the preferential incorporation of emotional waking-life experiences (Schredl, 2006; Malinowski and Horton, 2014; van Rijn et al., 2015), and concerns (Domhoff, 2003; Selterman et al., 2012) into dreams. This preferential incorporation can result in dream content being affected by psychopathology (Schredl and Engelhardt, 2001), acute mood and well-being (Chivers and Blagrove, 1999; Schredl, 2003), and major life circumstances such as pregnancy (Lara-Carrasco et al., 2013) and bereavement (Black et al., 2014). These continuities in the relationship between dream content and waking life experiences have led to the view that dreaming may be adaptive (Hartmann, 1995; Ruby, 2011), and one characteristic of adaptation that has been proposed for dreaming has been the ability to elicit insight (Maquet and Ruby, 2004; Cai et al., 2009). This proposal can be justified by the following three premises.

Firstly, that sleep, and in particular rapid eye movement (REM) sleep, is involved in functional reorganization of the brain that subserves emotional memory consolidation (Nishida et al., 2009; Dang-Vu et al., 2010; Wamsley and Stickgold, 2011; Groch et al., 2013, 2015), emotion regulation (Walker and Van der Helm, 2009), and cognitive insight (Wagner et al., 2004; Darsaud et al., 2011). During REM sleep (as compared to wakefulness), decreased activity in the dorsolateral prefrontal cortex and temporo-parietal junction, increased or maintained activity in the limbic system (notably amygdala, medial prefrontal/anterior cingulate, hippocampus, and parahippocampal cortex) and modification of functional connectivity between brain regions (Maquet et al., 1996, 2005; Nofzinger et al., 1997; Braun et al., 1998), may enable a different organization of cognition, favoring notably the triggering of emotional over neutral memories, the processing of spontaneous over actively selected thoughts, and an associative rather than a mainly logical mode of thinking.

Secondly, that functional reorganization and plasticity during sleep is reflected in dream content (Wamsley et al., 2010a,b; Wamsley and Stickgold, 2011), which may explain some famous claims of insight inspired by a dream (Maquet and Ruby, 2004; Cai et al., 2009). This fits with the suggestion of Perogamvros et al. (2013) that sleep and dreaming enable the offline reprocessing of emotions, associative learning, and exploratory behaviors, resulting in improved memory organization, waking life emotion regulation, social skills, and creativity.

Thirdly, and more speculatively, that the consideration of that dream content after waking can augment the associative and reorganizational processes that occur in sleep, and in wake. This premise has two components, both derived from the work of Freud (1953), that the process of free-association to the elements of a dream leads back to the precipitating sources of the dream (Baylor and Cavallero, 2001), and that the waking life free-associative process is similar to the flexible and creative cognitive processes suggested to be occurring during REM sleep (Walker et al., 2002).

### Assessing Insight Resulting From the Consideration of Dreams

The main empirical work on personal insight due to dream interpretation is that of Clara Hill, who uses one-to-one sessions with a therapist following Hill’s (1996). Exploration-Insight-Action model of dream interpretation. That work shows that personal insight from working with a recent dream is greater than when working with a report of a recent waking life event or with a dream of another person (Hill et al., 1993). However, that method is designed for use within psychotherapy, whereas our aim is to utilize methods designed for the lay (although skilled) use of considering dreams, in a group of people. For the current investigation, the Ullman and Schredl dream group methods were chosen, because they are designed for lay rather than psychotherapeutic use, because psychotherapy training is not needed for group leaders to run the sessions, and because there is published academic backing for the rationales for the two methods.

The Ullman (1996) dream group method aims at safe self-realizations and explorations rather than directed therapy. The procedure, detailed below, allows for the full describing of as much as can be remembered of the dream, the description by the dreamer of their recent waking life events and concerns before the dream, and the bringing together of these accounts of the dream and of waking life, so as to explore their connections. The Schredl dream group method (Schredl, 2007, 2011; Malinowski et al., 2014), also detailed below, aims to assist the dreamer in identifying common action and emotion patterns present in his or her dream and in his or her waking life. In both the Ullman and Schredl techniques, there is a phase in which the dream group can ask questions of the dreamer, in an attempt to consider connections between the content of the dream and the waking life experiences of the dreamer. In the Ullman technique, the focus is on the dreamer’s recent waking life experiences over the days prior to the dream, whereas in the Schredl technique the dreamer can consider associations to experiences from any time in his or her life.

### Control Conditions in Assessing Gains from Dream Interpretation

In their work on dream interpretation outcomes, Blagrove et al. (2010) and Edwards et al. (2013) did not have a control condition to which the dream condition would be compared. Those studies cannot, therefore, distinguish between outcomes specific to having a dream as the focus of the discussion, and outcomes due to the process of having a discussion irrespective of the initial text, if any, focussed on. In contrast, Hill et al. (2000) used a control condition of a description of a recent loss, finding that insights due to dream consideration were greater than due to loss consideration, and Hill et al. (1993) found greater insight from considering a dream than an event, although Diemer et al. (1996) found no difference between these. A further design used by Hill is the consideration by the dreamer of their own dream report, compared to the dreamer’s spouse considering the same dream (Kolchakian and Hill, 2002). For the two studies reported here a control condition of the consideration of a recent event is used, the event report then being subject to either the Ullman technique or the Schredl technique. The hypothesis is that insight outcomes will be greater for the dream than for the event condition, because the dream content reflects the associative state of the brain during sleep and/or the functional reorganization changes in sleep, and specifically REM sleep, as reviewed above. The modified state of the brain during sleep would thus explain the bizarreness component of dreams and also the important representation of emotions and retrieval of components which can be temporally remote episodic memories not recently accessed in waking life (Grenier et al., 2005). It is thus plausible that dreams might be able to bring to consciousness, either explicitly or after free associations, material that is important but currently not being considered in waking life. Of course, that any empathic conversation could do this is also a possibility, and one that is tested for by the use of the event discussion control condition.

The theoretical justification for choosing this control condition is derived from the work of Pennebaker. Pennebaker (1997) describes how the process of writing for 15–30 min per day for 3–5 days about an important emotional issue results in subjective and objective physical and mental health benefits (Baikie and Wilhelm, 2005; Lu and Stanton, 2010; Shim et al., 2011). Pennebaker describes two explanations for this effect. The first is that disclosure through writing removes inhibition, which is assumed to have been a source of stress. The second explanation, which Pennebaker supports in his 1997 paper, is that the process of writing causes cognitive changes, resulting in the development of a more coherent story about life concerns. As a comparison to writing about an important emotional issue, Pennebaker (1997) used a control condition of writing about a superficial issue, which was found to result in lower benefits. So as to provide an appropriate comparison to the dream condition the recent event provided by each participant for the two studies here was thus requested to be personally significant.

Edwards et al. (2013) differentiate between “aha” experiences that occur when the participant realizes what waking life event is the source of part of the dream content, and “aha” experiences that occur when the consideration of dream content produces some realization about one’s waking life, self, concerns, relationships, situations or actions. Both these types of aha experience contribute to the Exploration-Insight subscale on the GDI questionnaire. Edwards et al. (2013) show that Exploration-Insight is as high for the Ullman method as for the Hill method, which validates this dream group method, but did not differentiate in their results between these two types of insight. There is face validity to claiming that five items from the GDI Exploration-Insight and Action subscales refer to insight about oneself or one’s life rather than insight about the source of dream contents. The aims to report separately the mean of these five GDI items, and the mean of the five Gains from Event Interpretation (GEI) items, that specifically address insight about the self, and Gains from Event Interpretation (GEI) items that specifically address insight about the self.

Although the Ullman technique was found by Edwards et al. (2013) to be an effective procedure for establishing connections between dream content and recent waking life experiences, waking life sources were found for only 14% of dream report text. This was achieved in sessions lasting approximately 1 h. One aim of the current study is to extend this work by assessing the extent to which waking life sources can be identified in Ullman sessions that instead last 45 min, and to assess this measure for the Schredl dream group method also, and for the control waking life texts.

### Hypotheses

We hypothesize the following for the Ullman and Schredl studies:

(1) Participants will rate Exploration-Insight gains for the dream conditions as comparable to those obtained in the work of Hill, and specifically the meta-analysis results for that work as calculated by Edwards et al. (2013).
(2) Participants will rate Exploration-Insight gains more highly for the dream conditions than for the event conditions.
(3) Participants will rate the five questionnaire items that assess personal insight more highly for the dream conditions than for the event conditions.

## Materials and Methods

### Ullman Technique

Eleven participants (10 females, one male; ages 18–21, mean age = 20.18, SD = 0.98), all undergraduates at Swansea University, took part in this study. Each brought a written account of a recent dream and of a recent personally significant event to the group, which comprised the dreamer, and the authors CE and MB. The Ullman (1996) “Dream appreciation” method of detailing the content of the dream/event, detailing the recent waking life of the participant, and discussing or discovering connections between the report of the dream/event and prior waking life, was followed closely for the dream and the event texts.

The technique involves the following stages: 1A. Reading of the dream aloud by the dreamer. 1B. Clarification of the dream report by the group asking questions of the dreamer. 2A. Brief discussion of the dream by the group members other than the dreamer so as to imagine what feelings they would have experienced if the dream were their own, and then; 2B. Eliciting these individuals’ projections about the dream in terms of their own lives so as to give symbolic or metaphorical meaning to the dream images. 3A. Response by the dreamer to stage 2. 3B.1 Description by the dreamer of his/her waking life context for the dream, in terms of the dreamer’s life experiences, with particular emphasis on recent experiences and concerns. 3B.2 Reading the dream back to the dreamer, in the second person, so that any additional information about the dream or waking life can be obtained; and 3B.3 Orchestration, in which all members of the group suggest connections between information that the dreamer has given about their dream and information the dreamer has given about the dreamer’s life. For a full description of the process, see Ullman (1996).

### Schredl Technique

Nine participants (seven females, two males; ages 19–40, mean age = 27.11, SD = 9.09) took part in this study. Eight were current students at the University of Bedfordshire, one had completed postgraduate study. Each brought a written account of a recent dream and of a recent personally significant event to the group, which comprised the dreamer, author JM, and one of two research assistants. Schredl’s (2011) “Listening to the dreamer (LTTD)” method of detailing the content of the dream/event, and discussing or discovering patterns of behavior or emotion that are in common between the dream/event report and prior waking life, was followed closely for the dream and the event texts.

The first five stages of Schredl’s LTTD technique were used, as follows. 1. The dreamer shares a dream. The other group members ask questions with the aim of helping the dreamer to re-connect with the dream experience and to allow the dreamer to disclose more details about the dream. 2. The dream group ask the dreamer questions about whether they can make any associations between waking life memories and the dream. 3. The dreamer is asked to summarize the action pattern and emotion pattern of the dream. The actions and emotions are described in a very basic form at this point in the procedure. 4. The dreamer is asked to consider how the “Basic Action” and “Basic Emotion” patterns might link to sequences in waking life. 5. The dreamer is asked to consider whether he or she would like to alter any of their own thoughts or actions in the dream. (Schredl’s LTTD technique does allow for consideration of future cognitive and behavioral changes as a result of the dream, but this sixth stage was not included in the use of the technique in this study.)

The Ullman study received ethics approval from the Research Ethics Committee of the Department of Psychology, Swansea University. The Schredl study received ethics approval from the Research Ethics Committee of the Department of Psychology, University of Bedfordshire. Participants gave written informed consent to take part after being given full information about what was involved in taking part. Information was given for consulting clinically qualified well-being services in the event of distress or discomfort as a result of reporting or discussing dreams or events, and it was made clear to participants throughout the study that they could halt their involvement, or halt discussion of any matter, at any point without needing to give explanation.

### Measures

After the dream and event interpretation sessions participants completed the 14 item GDI questionnaire (Heaton et al., 1998b) and its counterpart the GEI questionnaire.

The GDI Exploration-Insight subscale comprises items on the experience of being in the group session, on insight obtained during the session about oneself or one’s life, and insight about memory sources for the dream. Some items refer to more than one of these three categories. The GDI’s Exploration-Insight subscale items are as follows (numbers refer to item number on the GDI questionnaire):

1. I was able to explore my dream thoroughly during the session.
2. I learned more about what this dream meant for me personally during the session.
6. I learned more from the session about how past events influence my present behaviour.
7. I learned more about issues in my waking life from working with the dream.
8. I felt like I was very involved in working with the dream during the session.
12. I learned things that I would not have thought of on my own.
13. I was able to make some connections, that I had not previously considered, between images in my dream and issues in my waking life.

The GEI questionnaire is an amended version of the GDI, with event substituted for dream throughout. For example, item 7, “I learned more about issues in my waking life from working with the dream” is changed to “I learned more about issues in my waking life from working with the event.” The GDI and GEI questionnaires each have 14 items and use a 9-point scale for each item (1–9, where 1 = “strongly disagree” and 9 = “strongly agree”), which results in three subscales: Exploration-Insight gains, Experiential gains, and Action gains. Each of the subscales has a range of scores of 1–9.

The Action gains subscale of the GDI and GEI has five items, which refer to being able to change bad dreams (or change waking life events) and change waking life cognitions or actions, as a result of the session. Three items on the Action gains subscale refer to personal insight:

5. I got ideas during the session for how to change some aspect(s) of myself or my life.
10. I learned a new way of thinking about myself and my problems.
11. I will use the things that I learned in this dream [event] interpretation in my life.

One aim of this paper is to investigate the use of a personal insight subscale comprising items 5, 6, 7, 10, and 11.

The third subscale of the GDI/GEI, the Experiential gains subscale, has two items concerning re-experiencing the dream [event] and its emotions in the session.

### Procedure

As there are cultural and historical expectations of hidden meanings and insight benefits from examining dreams (Morewedge and Norton, 2009), it is necessary to provide information to participants to justify examining an event. For the event condition justification a summary was given of the work of Pennebaker on the health and wellbeing benefits of constructing and examining one’s own written narratives. On recruitment, and at the start of the session, participants were given two short written justifications about the usefulness of considering and discussing the content of a recent dream and of considering and discussing the content of a recent waking life event. Justifications were matched for length and included citations of the work of Hill for the dream condition and of Pennebaker for the event condition, citations in each case were to three academic papers and one book.

All participants took part in a dream and an event condition, each condition lasted approximately 45 min. The order of conditions was counterbalanced across participants. Each session was digitally voice recorded and later transcribed. The length of time of each session and the length of time spent on each stage of the Ullman and Schredl methods were calculated from the session transcripts so as to check whether the conditions differed on these variables. The emotional intensity and valance of the dream and event reports were rated by the participant so that any differences in these can be controlled for: a 1–7 hedonic scale was used where 1 = Very Pleasant, and 7 = Very Unpleasant. After the second of the two sessions for the Ullman study participants completed the GDI and GEI. For the Schredl study the GDI and GEI were completed at the end of their corresponding session.

The transcribed digital recordings of the sessions were used to produce an initial dream or event report, this being the report as stated in stage 1 of the Ullman and Schredl techniques. A canonical dream or event report was then produced, this being the initial dream or event report plus all additional or amended content of the report from the whole session. Two judges then assessed the transcripts of the dream and event conditions for both techniques so as to quantify the number of words of each canonical report for which prior waking life correspondences or sources were identified by the dreamer in the session. Inter-rater reliability for the number of words in the canonical report having, from the session transcript, a waking life source, were as follows: ρ= 0.59, *p* = 0.006 for dream reports and ρ= 0.63, *p* = 0.003 for event reports. The number of words that both judges agreed as having prior waking life correspondences identified by the dreamer in each session was then calculated. The percentage of words for each canonical report for which prior waking life correspondences or sources were identified was then also calculated.

## Results

### Ullman Study Results

The time spent on each stage are reported in Table 1 and compared for dream versus event condition so as to ascertain whether the conditions were treated the same in terms of length of discussion. Table 1 shows that the two conditions did not differ in time dedicated to each stage, except for stage 1, telling and clarifying the dream/event. Table 1 also shows that the two conditions did not differ in report valence, that initial dream reports were significantly longer than initial event reports, and canonical dream reports were longer than canonical event reports, but not significantly so. Canonical reports were significantly longer than initial reports for dreams [*t*(10) = 7.001, *p* < 0.001] and events [*t*(10) = 6.046, *p* < 0.001]. Using independent judge scores of the transcripts, the mean number of words in the canonical dream and event reports identified by the dreamer, in the session, as connected to prior waking life, did not differ significantly between the dream and event conditions. Expressing this number of words as a percentage of the canonical report length, participants identified waking life sources for 19.42% of canonical dream report content and 22.52% of canonical event report content.

**Table 1:** **Ullman method: valence and length in words of the initial dream or event report, time spent on each of the stages of the Ullman method, length of canonical report, and number of words in each canonical report connected, during the group session, to prior waking life, for the dream and event conditions**.

	**Dream *M* (SD)**	**Event *M* (SD)**	***t*(10)**	***P***
Valence	5.00 (1.34)	3.91 (1.92)	1.883	0.089
Length of initial report (number of words)	230.45 (63.56)	94.00 (38.07)	6.287	<0.001
Length of stage 1 (min)	11.05 (2.14)	8.41 (2.45)	2.636	0.025
Length of stage 2 (min)	5.77 (1.29)	5.41 (2.71)	0.398	0.699
Length of stage 3a (min)	0.77 (0.72)	0.91 (0.49)	–0.504	0.625
Length of stage 3b.1 (min)	17.91 (4.64)	13.86 (3.58)	2.01	0.073
Length of stage 3b.2 (min)	2.50 (0.71)	2.09 (0.58)	1.24	0.242
Length of stage 3b.3 (min)	6.73 (2.24)	5.95 (3.03)	0.866	0.407
Length of canonical report (number of words)	377.82 (121.46)	278.82 (115.05)	1.665	0.127
Number of words in canonical report connected to waking life	73.36 (17.31)	63.55 (25.91)	1.238	0.244

Table 2 shows that the dream condition was significantly higher than the event condition on the Exploration-Insight subscale, as hypothesized. Items 5, 6, 7, 10, and 11 of the GDI/GEI have a face validity of assessing level of Personal Insight obtained from the dream or event discussion. Pooling the GDI data from the current two studies and from Edwards et al. (2013), the five GDI items had a Cronbach’s alpha = 0.778, and the corresponding five items on the GEI from the current two studies had a Cronbach’s alpha = 0.893, indicating that the items can be taken together, and used as a measure of personal insight. Table 2 presents the means of the five items for the dream and event conditions, showing that the dream condition resulted in significantly higher ratings for Personal Insight than did the event condition, as hypothesized. Scores on the Action subscale did not differ significantly between conditions. Scores on the Experiential subscale and on GDI/GEI item 1 show that the dream and event reports were explored equally thoroughly during the sessions.

**Table 2:** **Gains from Dream Interpretation and Gains from Event Interpretation subscale scores for the Ullman dream and event conditions, mean of the personal insight items (5, 6, 7, 10, 11), and mean score for dream/event exploration item 1**.

	**Dream *M* (SD)**	**Event *M* (SD)**	***t*(10)**	***P***
Exploration-insight	7.82 (0.84)	7.21 (1.13)	3.59	0.005
Personal insight	6.60 (1.43)	6.20 (1.58)	2.29	0.045
Action	5.84 (1.38)	5.87 (1.65)	–0.15	0.882
Experiential	6.55 (1.37)	6.55 (1.62)	0.00	1.000
Item 1^a^	8.18 (1.25)	7.55 (1.92)	1.10	0.295

a Item 1, “I was able to explore my dream/event thoroughly during the session.”

The meta-analysis of the work of Clara Hill, calculated by Edwards et al. (2013), showed the following GDI subscale means: Exploration-Insight gains, mean = 7.40 (SD = 1.15); Experiential gains, mean = 7.03 (1.56); Action gains, mean = 6.51 (1.34). The GDI subscale means here do not differ significantly from the results of Hill for the three subscales [*t*(10)s = 1.66, –1.16, –1.61, for Exploration-Insight, Experiential, and Action subscales, respectively].

### Schredl Study Results

The time spent on each stage are reported in Table 3 and compared for dream versus event condition so as to ascertain whether the conditions were treated the same in terms of length of discussion. Table 3 shows that the two conditions did not differ in time dedicated to each stage, except for stage 2, where the group ask the dreamer questions about whether they can make any associations between the dream or event and prior waking life memories. Table 3 also shows that the two conditions did not differ in report valence, that initial dream reports were significantly longer than initial event reports, and canonical dream reports were longer than canonical event reports, but not significantly so. Canonical reports were significantly longer than initial reports for dreams [*t*(8) = 4.599, *p* = 0.002] and events [*t*(8) = 4.729, *p* = 0.001]. Using independent judge scores of the transcripts, the mean number of words in the canonical dream and event reports identified by the dreamer, in the session, as connected to prior waking life, did not differ significantly between the dream and event conditions. Expressing this number of words as a percentage of the canonical dream length, participants identified waking life sources for 17.26% of the text of canonical dream reports and 17.18% of the text of canonical event reports.

**Table 3:** **Schredl method: valence, and length in words of the initial dream or event report, time spent on each of the stages of the Schredl method, length of canonical report, and number of words in each canonical report connected, during the group session, to prior waking life, for the dream and event conditions**.

	**Dream *M* (SD)**	**Event *M* (SD)**	***t*(8)**	***P***
Valence	5.11 (1.83)	4.89 (2.32)	0.308	0.766
Length of initial report (number of words)	214.89 (105.58)	92.56 (41.16)	3.579	0.007
Length of stage 1 (min)	8.44 (2.95)	8.33 (2.80)	0.074	0.943
Length of stage 2 (min)	10.06 (4.44)	5.89 (2.19)	2.921	0.019
Length of stage 3 (min)	4.67 (1.48)	4.28 (2.90)	0.426	0.681
Length of stage 4 (min)	3.39 (1.93)	3.44 (3.02)	–0.084	0.935
Length of stage 5 (min)	1.56 (1.01)	1.50 (1.00)	0.109	0.916
Length of canonical report (number of words)	458.33 (173.38)	355.00 (184.97)	1.876	0.097
Number of words in report connected to waking life	79.11 (22.74)	61.00 (24.73)	1.950	0.087

Table 4 shows that the dream condition was higher than the event condition on the Exploration-Insight subscale and on Personal Insight, but not significantly so. Scores on the Action subscale did not differ significantly between conditions. Scores on the Experiential subscale and on GDI/GEI item 1 show that the dream and event reports were explored equally thoroughly during the sessions.

**Table 4:** **Gains from Dream Interpretation and Gains from Event Interpretation subscale scores for the Schredl dream and event conditions, mean of the personal insight items (5, 6, 7, 10, 11), and mean score for dream/event exploration item 1**.

	**Dream *M* (SD)**	**Event *M* (SD)**	***t*(8)**	***P***
Exploration-insight	7.83 (1.09)	7.44 (1.56)	1.55	0.159
Personal insight	6.69 (1.63)	6.36 (1.86)	0.66	0.527
Action	6.62 (1.64)	6.53 (1.62)	0.25	0.809
Experiential	7.89 (1.58)	7.44 (1.63)	0.84	0.426
Item 1^a^	8.67 (1.00)	8.44 (1.13)	0.61	0.559

a Item 1, “I was able to explore my dream/event thoroughly during the session.”

The GDI subscale means here do not differ significantly from the results of Hill for the three subscales from the Edwards et al. (2013) meta-analysis [*t*(8)s = 1.18, 1.63, and 0.20, for Exploration-Insight, Experiential, and Action subscales respectively].

## Discussion

In accordance with the first hypothesis, participants gave ratings on Exploration-Insight gains for the dream conditions that were comparable to those obtained in the work of Hill. Action and Experiential gains for the dream conditions were also comparable to those obtained in the work of Hill. Regarding the second hypothesis, the Ullman dream discussion condition resulted in significantly higher Exploration-Insight scores than did the event discussion control. In accordance with the third hypothesis, that Personal Insight would be greater after considering dreams than considering events, Personal Insight gains as assessed by five items taken from the Exploration-Insight and Action subscales were significantly higher in the Ullman dream than event condition. For the Schredl dream condition self-ratings for Exploration-Insight and Personal Insight were very close to the Ullman group ratings, but did not exceed significantly the ratings from the Schredl event condition. For both techniques the Experiential gains results show no significant difference between the dream and event conditions, hence the event discussions were engaged with, to the extent of re-experiencing feelings and reliving the event, as highly as for the dream discussions. Item 1 of the GDI/GEI similarly shows that dreams and events were explored equally thoroughly in the sessions. In general, time spent in each stage was the same for the dream and event conditions in each technique, showing that the conditions were treated equally in this regard.

Participants identified waking life sources for 19.4% of dream content in the Ullman sessions and 17.3% of dream content in the Schredl sessions; in comparison, in Edwards et al. (2013), 14% of dream report text was found to be directly related to recent waking life sources. The corresponding figures for text of recent event reports being connected in the sessions to prior waking life sources are 22.5 and 17.2% for the Ullman and Schredl techniques respectively. This indicates that the majority of text in dream reports, and waking life event reports, does not have obvious correspondences with prior waking life experiences, as least as can be identified in a 45 min discussion. Importantly, whereas the Ullman dream and event conditions differed significantly on Exploration-Insight and Personal Insight, they did not differ on the number of words of the dream or event report that were related by the dreamer to waking life during the sessions. The difference in insight outcomes between the conditions is thus not due to a confound of amount of waking-life relevant text in the reports. The results for the Schredl technique that there was no significant difference in eliciting personal insight for dream and event reports can be seen in terms of the Dodo effect, where there are equally high outcomes between different psychotherapeutic techniques and theories (Luborsky et al., 1975; Wampold et al., 1997). Indeed in practical terms it is not necessary for the dream condition to exceed the event condition in gains for benefits to be claimed for the group interpretation of dreams, even though theoretical reasons for a difference in outcomes between conditions can be proposed and hypotheses for this tested. The personal insight scores for event discussion in the two studies (means = 6.20 and 6.36 on a 1–9 scale) do indicate benefits to writing down and discussing an account of a recent significant event, and are thus supportive of Pennebaker’s model of benefits of writing-based expression. However, it may be argued that higher scores could be obtained from other control conditions, with the relative advantage of the dream condition not then being so apparent. One possibility for a different control condition is the use of a recent daydream, to which the Ullman and Schredl techniques could then be applied. A method for the successful collection of daydream reports is described by Noreika et al. (2010), and used by them as a comparison condition for dream reports. As the daydream content is influenced by the current waking life concerns of the participant, this would be a suitable control condition in future studies. Furthermore, as REM sleep characteristics provide the theoretical basis for the hypothesis of greater insight following dream than event discussions, non-REM (NREM) dream reports could also be used as a control to which REM dream reports are compared.

The hypothesis that consideration of dream content might result in personal insight was supported by the premise that dreaming reflects emotional memory consolidation, functional reorganization and neural processes of insight occurring during sleep. However, it is not necessary to posit that dreams are related to a function of sleep for there to be a hypothesis of benefits in the consideration of dream content. It is possible that the sleeping brain state, for non-necessarily functional reasons, allows dream content to represent waking life matters about which we are more normally defended or even unaware when awake, because active inhibition of attending to these is suppressed during sleep (Freud, 1953; Wegner et al., 2004; Erdelyi, 2006; Bryant et al., 2011). To distinguish functional from non-functional accounts of dreaming would require investigations of the cognitive or other effects, if any, of unrecalled dreams, in addition to recalled dreams as in the current study: due to the difficulties involved such studies have as yet not been performed. Furthermore, Blagrove (2011) cautions that studies that do not experimentally alter dream content are insufficient to demonstrate that characteristics or effects of dream content are functional, as opposed to being solely reflective of pre-sleep emotions, cognitions and experiences (the latter view is argued by De Koninck et al., 2012), although possible higher level socio-cognitive characteristics of dream content, and possibly REM sleep processes (Blagrove et al., 2013) do need to be considered. In addition, we acknowledge also that there is the view, discussed in van Rijn et al. (2015), that declarative memory consolidation is primarily a function of Slow Wave Sleep rather than REM sleep (Diekelmann and Born, 2010; Lewis and Durrant, 2011). Such a view leads to the conclusion that the study of home dream content that is likely to be from REM sleep, as in the current study, might not have implications for the understanding of sleep-related functional memory processes, although studies referred to in the introduction of the current paper would counter this view. To investigate this further, the studies here should be repeated with REM and NREM dreams separately, with the hypothesis that there will be greater insight elicited from the former.

The two studies had various limitations. It may be that the types of insights elicited differ between the dream and event conditions, just as Hill et al. (2000) found that dream elicited insights tended to refer to personal relationships whereas consideration of loss insights tended to relate to the connection between past and present experience. Future work should address whether there are such qualitative differences. It may also be that full benefits from dream or event discussions do not occur for the brief timescale of discussion and assessment used in the current studies, and that further gains might occur with time. Furthermore, these lay group results might underestimate gains that could occur with more experienced researchers running the groups, or researchers with clinical experience, or participants with greater experience of considering their own dreams. It may also be that, as suggested by Heaton et al. (1998a), particular types of dreams, such as troubling or recurrent ones, are more important to explore, whereas the current studies used the most recent dream of each participant irrespective of content or type. Lastly, the sample sizes for the current studies were small, and, as most participants were female it is not clear whether these results would be generalizable to males, either because of differences between males and females in attitude toward dreams and dream interpretation (Schredl and Piel, 2008), or because of differences in dream content of males and females (Domhoff, 1996, 2003).

The extent to which the current results generalize to psychotherapy involving clients with a psychopathology needs also to be addressed and is dependent on several considerations. The first is the difference in dream content from that of controls that can result from particular psychopathologies. For example, in depression, compared to controls, themes of death (Firth et al., 1986; Schredl and Engelhardt, 2001), hostility and masochism (Hauri, 1976), and a lower number of characters (Barrett and Loeffler, 1992). In dissociative disorder, a high level of nightmares (Agargun et al., 2003). In schizophrenia, greater dream bizarreness (Noreika et al., 2010). In personality disorder, dreams with a lower number of interactions and higher emotionality than for controls (Guralnik et al., 1999). In terms of Pesant and Zadra’s (2004) conceptualization that working with dreams in therapy can result in various benefits, such as client insights, increased involvement of the client in the therapeutic process, promoting a safe and trusting environment, facilitating access to issues that are central to the clients’ lives, and a better understanding of clients’ dynamics and clinical progress, benefits in some of these areas might be obtained for individuals with psychopathology. There is as yet, though, only a limited literature on this: Pesant and Zadra (2004) consider the extension of therapeutic consideration of dream content to psychopathological conditions, as also do Heaton et al. (1998a) in a case study on the analysis of recurrent and non-recurrent dreams of a client with dissociation. Secondly, a confounding factor here is the association of psychopathology with nightmares (Kales et al., 1980; Levin and Raulin, 1991; Berquier and Ashton, 1992; Levin, 1998; Chivers and Blagrove, 1999; Levin and Fireman, 2002) and with trait nightmare distress, which is the adverse reaction to having nightmares (Belicki, 1992; Claridge et al., 1997, 1998). Nightmares have been addressed in cognitive therapy, with the aim of reducing nightmare frequency using imagery rehearsal (Krakow et al., 2000; Lu et al., 2009; Ulmer et al., 2011), but not for any psychotherapeutic use of the nightmare content. As the studies here did not have nightmares reported, nor individuals reporting any psychopathology, the generalizability of the results to individuals with those characteristics requires further investigation. Furthermore, regarding client insight resulting from dream discussion, condition severity, which is inversely related to client insight toward a condition (Trevisi et al., 2012), might be a modulating variable for GDI.

Whereas the main aim of the current studies was to assess differences in gains between talking about a dream and talking about an event, it is important to recognize that some people might wish to talk about their dreams for reasons other than obtaining informational benefit from doing so. This point is reinforced by our findings that the benefit of considering dreams rather than events was significant but small in one study, and non-significant in the other. It is thus important to investigate the various reasons that people might wish to tell or discuss their dreams, which may be a function of the puzzling or emotional nature of the content. In this regard the finding by Hill et al. (2013) should be noted, that, although clients in psychotherapy who work with dreams do report that the dream work was helpful, session outcomes are not greater than for clients who do not discuss dreams in therapy. Of relevance are the findings of Olsen et al. (2013), who show a significant, positive correlation between dream sharing frequency in couples and perceived relationship intimacy, but with the benefit of dream sharing being primarily as entertainment. The study of insight benefits of dream sharing should thus be augmented by the study of the various motivations and consequences for sharing dreams, and individual differences in these.

To summarize, An impactful consequence of recalling dreams has been shown by Wright et al. (2014), who found that many recently bereaved persons experience vivid and deeply meaningful dreams of the deceased that may reflect and impact the process of mourning. Such dreams affect the bereavement process, including levels of comfort, sadness, and acceptance of the loved one’s death. The authors conclude by emphasizing the importance for grief counselors of awareness of working with dreams. Our results support the view that benefits can be obtained by such consideration of dream content, in terms of identifying the waking life sources of dream content, and because personal insight gains may also occur. Mechanisms for this should now be investigated by the comparison of dream group outcomes for REM and NREM dreams separately.

### Conflict of Interest Statement

The authors declare that the research was conducted in the absence of any commercial or financial relationships that could be construed as a potential conflict of interest.
